# Feasibility of the Participation and Activity Inventory for Children and Youth (PAI-CY) and Young Adults (PAI-YA) with a visual impairment: a pilot study

**DOI:** 10.1186/s12955-017-0677-x

**Published:** 2017-05-11

**Authors:** Ellen Bernadette Maria Elsman, Ruth Marie Antoinette van Nispen, Gerardus Hermanus Maria Bartholomeus van Rens

**Affiliations:** 1 Department of Ophthalmology, VU University Medical Centre and the Amsterdam Public Health research institute, PO Box 7057, 1007 MB Amsterdam, The Netherlands; 2 Department of Ophthalmology, Elkerliek Hospital, Wesselmanlaan 25, 5707 HA Helmond, The Netherlands

**Keywords:** Visual impairment, Children, Adolescents, Young adults, Pilot study, Participation and Activity Inventory (PAI)

## Abstract

**Background:**

Having a visual impairment affects quality of life, daily functioning and participation. To assess rehabilitation needs of visually impaired children and young adults, the Participation and Activity Inventory for Children and Youth (PAI-CY) and Young Adults (PAI-YA) were developed. The PAI-CY comprises four questionnaires for different age categories: 0–2 years, 3–6 years, 7–12 years and 13–17 years. This pilot study assesses the feasibility and acceptability of the PAI-CY and PAI-YA, and the relevance of the content of the questionnaires.

**Methods:**

In addition to the regular admission procedure, the PAI-CY and PAI-YA were completed by 30 participants (six per questionnaire). For the PAI-CY, parents completed the questionnaire online prior to admission. From age 7 years onwards, children completed the questionnaire face-to-face with a rehabilitation professional during the admission procedure. Young adults completed the PAI-YA online. Subsequently, participants and professionals administered an evaluation form.

**Results:**

Overall, 85% of the parents rated all aspects of the PAI-CY neutral to positive, whereas 100% of all children and young adults were neutral to positive on all aspects, except for the duration to complete. The main criticism of professionals was that they were unable to identify actual rehabilitation needs using the questionnaires. Minor adjustments were recommended for the content of questions.

**Conclusions:**

Parents, children and young adults were mostly satisfied with the questionnaires, however, professionals suggested some changes. The adaptations made should improve satisfaction with content, clarification of questions, and satisfaction with the questionnaires in compiling a rehabilitation plan. Although face and content validity has been optimized, a larger field study is taking place to further develop and evaluate the questionnaires.

## Background

Although no accurate prevalence data are available on visually impaired children aged 0–17 years and young adults aged 18–25 years in the Netherlands, visual impairments are estimated to affect 2600 children aged 0–14 years [1]. In the Netherlands, most visual impairments in children and young adults are due to developmental and (rare) genetic disorders, with the most common diagnoses being cerebral visual impairment, albinism and nystagmus [2]. Visual impairments impact the participation of children and young adults [3–5] as well as their daily functioning and quality of life [6, 7]; these impairments can also cause social isolation which (over time) can lead to lowered self-esteem and depression [8, 9]. Since there is no general consensus on the definition of participation in children and those with an impairment [10], patient record studies were conducted in order to evaluate the rehabilitation goals of children and young adults with a visual impairment [11, 12]. These studies found that challenges most frequently faced by children are related to learning and applying knowledge, mobility, and major life areas (although goals related to other participation domains may be underrepresented) [12]. Young adults most frequently face challenges related to mobility, domestic life, communication, interpersonal interaction/relationships, general tasks/demands, major life areas and leisure activities [11]. Furthermore, concept-mapping studies have been conducted with children, parents and professionals from low vision rehabilitation centres to get more insight into the impact of a visual impairment on activities and participation [4, 5]. In children, it was found that low vision affects sensorial development and physical, psychological, and social well-being. However, external factors such as educational type and parental influence either facilitate or hinder participation. Furthermore, because of the rapid development of children, each life stage has different aspects which are most influenced by the visual impairment. For example, the attachment process most crucially influenced the first life years, whereas mobility and general development were most important for primary school-aged children [4]. Having a visual impairment as a young adult affects various life-aspects related to participation, including activities related to work, study, social skills and relationships, activities of daily living, leisure time and mobility. Especially activities related to study and work were considered important by young adults [5]. These studies also contributed to operationalizing the construct of participation for these populations further.

Low vision rehabilitation centres can help visually impaired children and young adults to identify their needs and become more self-reliant by offering rehabilitation. To assess rehabilitation needs of adults in an objective and systematic way, the Participation and Activity Inventory (PAI) was developed and implemented in the Netherlands [13, 14]. The PAI is based on the Activity Inventory of Massof et al. [15] and the nine domains of the Activity and Participation component of the International Classification of Functioning, Disability and Health (ICF) from the World Health Organization (WHO) [16]. The domains of the PAI are similar to the domains of the Activity and Participation chapter of the ICF, i.e. learning and applying knowledge; general tasks and demands; communication; mobility; self-care; domestic life; interpersonal interactions and relationships; major life areas; and community, social and civic life.

The content of the PAI focuses on the needs of adults and is, therefore, not applicable to identify the needs of children aged 0–17 years, since needs develop with increasing age [4, 12]. Moreover, the life stage of young adults aged 18–25 years is characterized by the transition of becoming an adult, with a growing need for independence and autonomy [17], making the extensive content of the PAI less applicable. Because multidisciplinary rehabilitation centres currently lack an instrument to identify the needs of children and young adults, they are dependent on their own knowledge and expertise when creating an inventory of needs. Relying solely on the personal expertise of low vision professionals (e.g. social workers, occupational therapists, educational and developmental psychologists) carries a risk of bias and an underrepresentation of needs [11, 18]. Furthermore, incorrect identification of rehabilitation needs might influence referral to rehabilitation programmes and the quality of care provided [19].

Therefore, we developed PAI questionnaires to assess the needs of visually impaired children and youth aged 0–17 years (PAI-CY), and young adults aged 18–25 years (PAI-YA). To aid interpretation of the PAI-CY, four different age categories were formed based on the WHO criteria: 0–2 years (infants and toddlers), 3–6 years (preschool children), 7–12 years (school-aged children), and 13–17 years (adolescents). For each age category, a different PAI-CY was developed, which differs in the domains covered and the number of items included.

An important step in the ongoing development, validation and implementation of the PAI-CY and PAI-YA is to perform a pilot study [20]. The present pilot study is part of a larger validation study, and aims to assess the feasibility and acceptability of the PAI-CY and PAI-YA in the regular admission procedure: i.e. to evaluate whether use of the questionnaires in practice is workable and whether the questionnaires are acceptable to both clients and professionals. Another aim is to determine whether relevant topics are included and whether all items and response categories are clear. For this purpose, clients and rehabilitation professionals were asked for their perceptions and experiences after using the PAI-CY and PAI-YA.

## Methods

### Development of the PAI-CY and PAI-YA

The most important stakeholders were involved in the development of the PAI-CY and the PAI-YA, thereby largely contributing to the content and face validity of the questionnaires.

For the PAI-CY these were professionals of low vision rehabilitation centres, and parents of children aged 0–17 years and children aged 7–17 years. For the development of the PAI-YA, professionals of low vision rehabilitation centres and young adults aged 18–25 years were involved. The most suitable method for each stakeholder was chosen: parents received an online questionnaire, focus group discussions and semi-structured interviews were held with children aged 7–17 years, and concept-mapping workshops were organised for professionals and young adults aged 18–25 years. All methods aimed at generating statements regarding activities a child/young adult with a visual impairment would like to participate in. For the PAI-CY, the results from questionnaires, focus groups, interviews and concept-mapping were combined in a conceptual model, which contributed to the development of the PAI-CY questionnaires [4]. For the PAI-YA, concept-maps of professionals and young adults, and the combined concept-map, contributed to the development of the PAI-YA questionnaire [5].

The PAI-CY and PAI-YA consist of activities divided into different domains, e.g. play, self-reliance, mobility, communication, social relationships, day-care/school/study, leisure time and acceptance/self-consciousness (see Table 2 for a complete overview of the domains per questionnaire). Each item is scored on a 4-point scale with the response options: not difficult (0), slightly difficult (1), very difficult (2), impossible (3). The response options ‘Not applicable’ and ‘I don’t know’ were treated as a missing value.

### Participants

Participants with a visual impairment were recruited from two low vision rehabilitation centres in the Netherlands, Royal Dutch Visio and Bartiméus. All participants met the study criteria when they were aged between 0 and 25 years and fulfilled the criteria for visual impairment according to the WHO [21]. Furthermore, participants were considered to meet the study criteria when they were eligible for care in a low vision rehabilitation centre according to Dutch rehabilitation referral guidelines, i.e. when the visual impairment caused limitations in activities of daily living that could not be solved by regular healthcare services [22]. In addition to the study criteria above, participants had to have sufficient knowledge and understanding of the Dutch language and adequate cognitive abilities.

The study protocol was approved by the Medical Ethical Committee of the VU University Medical Centre, Amsterdam. This study was performed in accordance with the ethical standards as laid down in the Declaration of Helsinki and its later amendments. Informed consent was obtained from all participants, including children aged 13 years and older and their parents. Furthermore, informed consent was obtained from parents of children below the age of 13 years.

### Data collection

The PAI-CY was administered as part of the regular admission procedure of low vision rehabilitation centres. Parents with a child in one of the age categories of the PAI-CY completed the corresponding questionnaire online in advance of the admission procedure by the rehabilitation service. Children aged 7–17 years completed the corresponding questionnaire face-to-face with a rehabilitation professional of a low vision rehabilitation centre during the admission procedure. Immediately after assessing the PAI-CY, parents and children were asked to fill in an evaluation form about the PAI-CY and its assessment, aimed at determining and improving acceptability and feasibility of the PAI-CY. The professional involved in the admission procedure was also asked to complete an evaluation form about the PAI-CY for the questionnaire filled in by parents, and, if applicable, about the questionnaire filled in by children. Due to a longer than expected inclusion period, the first author also administered the PAI-CY to a small number of participants; these participants were already enrolled in care from low vision rehabilitation centres. Children were visited at their home to administer the PAI-CY, whereas parents completed the questionnaire online.

The PAI-YA was also tested with young adults who were already enrolled in care from low vision rehabilitation centres; they also completed the PAI-YA and evaluation form online. Parents and young adults filled in questions about demographic characteristics, such as date of birth (of their child), sex (of their child), nationality, and educational level. In addition, parents were asked which parent administered the questionnaire e.g. mother, father or together, their marital status and whether their child had siblings. Participants’ clinical characteristics, such as visual acuity and diagnosis were retrieved from their medical file. All questionnaires were entered in an internet-based questionnaire programme where no missing values were allowed.

### Statistical analysis

Descriptive statistics were calculated for demographic variables of participants, including age, gender, information regarding eye condition and visual acuity, and which parent filled in the questionnaire on behalf of the parents. Responses to the PAI-CY and PAI-YA were examined to indicate floor and ceiling effects. Floor and ceiling effects were considered present when at least five out of six participants rated the lowest or highest score possible, respectively (i.e. ‘not difficult’ and ‘impossible’). Furthermore, mean difficulty scores were calculated per domain and age category, using the mean domain difficulty for each respondent. The mean domain difficulty for each respondent was calculated when at least 50% of the items in that domain were scored. Data were analysed using SPSS version 22.0.

Furthermore, satisfaction regarding the PAI-CY and PAI-YA was assessed using the evaluation forms of parents, children and professionals. If possible, qualitative data from the evaluation forms which were similar were combined. Based on the results of this study, each item of the PAI questionnaire was discussed and consensus between the first and the second author was reached in order to develop an improved version of the PAI-CY and PAI-YA.

## Results

### Participant characteristics

A total of 30 respondents participated in this study. Each PAI-CY was filled in by six parents of children with a visual impairment. Simultaneous measurements were taken from 12 children in the age categories 7–12 years and 13–17 years. For the age categories 0–2 years, 3–6 years, 7–12 years and 13–17 years, five, six, four and one parent (and child), respectively, completed the PAI-CY as part of the regular admission procedure. All other participants did not complete the PAI-CY as part of their regular admission, as they were already in care at low vision rehabilitation centres, and questionnaires were administered by the researcher instead.

In total, 17 evaluation forms were available from professionals, as four of them did not compete (both) evaluation forms. Subsequently, six young adults who were already receiving care filled in the PAI-YA. Table 1 presents characteristics of the participants. Ophthalmic diagnoses are classified according to eye structure affected by the disease because there was a large variation in ophthalmic diagnoses. Examples of cerebral diagnoses are cerebral visual impairment and cerebral palsy, whereas examples of retinal diagnoses are hyperopia, retinitis pigmentosa and Stargardt’s disease.

**Table 1:** Characteristics of the participants by age group

	PAI-CY 0–2	PAI-CY 3–6	PAI-CY 7–12	PAI-CY 13–17	PAI-YA 18–25
Age in years					
Mean (range)	19.3 (8–30) ^a^	4.5 (3–6)	9.8 (8–12)	15 (13–17)	22 (18–26)
Sex					
Male, N (%)	3 (50)	5 (83.3)	4 (66.7)	5 (83.3)	1 (16.7)
Visual acuity					
Blind, N (%) ^b^			1 (17%)	1 (17%)	1 (17%)
Low vision, N (%)	3 (50%)	3 (50%)	5 (83%)	5 (83%)	5 (83%)
Unknown, N (%)	3 (50%)	3 (50%)			
Ophthalmic diagnosis					
Cerebral, N (%)	2 (33.3%)	2 (33.3%)	1 (16.7%)	1 (16.7%)	1 (16.7%)
Retinal, N (%)		1 (16.7%)	5 (83.3%)	3 (50%)	4 (66.7%)
Lens, N (%)	1 (16.7%)				
Optic, N (%)				1 (16.7%)	1 (16.7%)
Unknown, N (%)	3 (50%)	3 (50%)		1 (16.7%)	
Proxy parents					
Mother, N (%)	2 (33.3%)	4 (66.7%)	6 (100%)	4 (66.7%)	
Father, N (%)	2 (33.3%)	1 (16.7%)		1 (16.7%)	
Together, N (%)	2 (33.3%)	1 (16.7%)		1 (16.7%)	
Duration in minutes					
Mean (range)	13.3 (5–25)	21.7 (15–45)	16.7 (5–25)	14.2 (10–20)	45.8 (30–90)

^a^Age in months instead of years

^b^Blind: visual acuity ≤ 0.05 and/or visual field ≤ 10°

### Responses to the PAI-CY and PAI-YA

Response category frequencies were calculated for each questionnaire. Only floor effects were found and these were present in all questionnaires. In the PAI-CY 0–2 year, floor effects were present in only one item (2%) related to raising the head. In the PAI-CY 3–6 years, floor effects were present in 25% of the items; these were related to various domains and tasks, e.g. recognising facial expressions, talking, recognising colours, and eating independently. Floor effects were present in 12 and 16% of the items in the PAI-CY 7–12 years administered by parents and children, respectively. Floor effects of parents and children partly overlapped and were, for example, found in participating in group activities, playing imaginary games, telling your parents about activities, finding your way in school, going to the bathroom, and actively participating in a conversation. Floor effects were found in 6 and 20% of the items in the PAI-CY 13–17 years administered by parents and children, respectively, which also partly overlapped. Floor effects were found, for example, in using social media, searching for information, doing the dishes, and asking acquaintances for help. In the PAI-YA 18–25 years, floor effects were found in 30% of the items, e.g. finding visual aids, cooking, managing finances, taking medication, using the computer, inviting friends, dating, planning a daytrip, listening to music, and expressing feelings.

Further assessment of the mean difficulty (overall possible range: 0–3) showed that domains in the PAI-CY 0–2 years ranged from 0.6 to 1.5 in difficulty, and domains in the PAI-CY 3–6 years ranged from 0.1 to 1.5 in difficulty. According to parents, domains in the PAI-CY 7–12 years ranged from 0.4 to 1.1 in difficulty while according to children difficulty ranged from 0.3 to 0.9. The mean difficulty of domains in the PAI-CY 13–17 years ranged from 0.6 to 1.1 according to parents and 0.2–0.8 according to children. On average, children rated the difficulty of all domains lower than their parents, except for the domains finances and leisure time by children aged 7–12 years. Mean difficulty of domains in the PAI-YA 18–25 years ranged from 0.1 to 0.8. Table 2 presents the most important characteristics of the domains in each questionnaire.

**Table 2:** Domain characteristics for each PAI questionnaire

Domain in PAI-CY/PAI-YA	PAI-CY 0–2					PAI-CY 3–6					PAI-CY 7–12						PAI-CY 13–17						PAI-YA 18–25				
	No. of items pilot PAI	Domain order pilot PAI	Mean difficulty parent (SD)	No. of items final PAI	Domain order final PAI	No. of items pilot PAI	Domain order pilot PAI	Mean difficulty parent (SD)	No. of items final PAI	Domain order final PAI	No. of items pilot PAI	Domain order pilot PAI	Mean difficulty parent (SD)	Mean difficulty child (SD)	No. of items final PAI	Domain order final PAI	No. of items pilot PAI	Domain order pilot PAI	Mean difficulty parent (SD)	Mean difficulty child (SD)	No. of items final PAI	Domain order final PAI	No. of items pilot PAI	Domain order pilot PAI	Mean difficulty young adult (SD)	No. of items final PAI	Domain order final PAI
Bonding	6	1	0.8 (0.9)	6	1	5	1	0.3 (0.2)	5	1																	
Incentive processing	4	2	1.0 (0.9)	4	2	3	2	0.4 (0.3)	4	2																	
Visual attention	4	3	1.2 (1.1)	5	3	4	3	0.8 (0.4)	4	3																	
Sensorial functioning	12	8	1.5 (0.8)	13	8	10	11	1.5 (0.8)	10	13																	
Orientation	2	4	1.3 (1.3)	2	4	3	4	0.7 (0.5)	3	4																	
Motor functioning						2	7	1.3 (0.6)	2	8																	
Reading and writing									5	11																	
Play	5	5	1.1 (1.0)	5	5	3	5	0.7 (0.8)	3	6	3	1	0.6 (0.5)	0.6 (0.4)	3	1											
Self-reliance						4	10	0.8 (0.5)	4	12	5	7	0.6 (0.5)	0.3 (0.3)	5	7	7	6	0.6 (0.7)	0.2 (0.3)	10	6					
Finances											1	9	0.4 (0.5)	0.7 (0.8)	1	9	1	8	0.7 (1.2)	0.3 (0.8)	1	8					
Mobility	7	6	0.6 (0.9)	7	6				6	5	4	3	1.0 (0.4)	0.8 (0.7)	4	3	7	1	1.1 (1.2)	0.8 (0.8)	7	2	14	5	0.7 (0.4)	16	2
Communication	2	7	0.8 (1.3)	2	7	5	8	0.1 (0.2)	4	9	12	5	0.7 (0.3)	0.6 (0.5)	12	5	11	4	0.8 (0.8)	0.4 (0.4)	11	4	11	13	0.3 (0.2)	11	12
Social relationships						6	6	1.1 (0.6)	6	7	6	2	0.5 (0.5)	0.3 (0.3)	6	2	7	3	0.9 (0.8)	0.5 (1.0)	7	3	11	7	0.5 (0.3)	11	9
Day-care/school/study						6	9	0.9 (0.4)	6	10	11	6	0.9 (0.4)	0.6 (0.6)	11	6	9	5	0.8 (0.4)	0.5 (0.4)	9	5	8	15	0.7 (0.4)	8	14
Leisure time											3	4	0.5 (0.4)	0.7 (0.9)	8	4	4	2	1.0 (1.0)	0.6 (0.5)	9	1	11	11	0.3 (0.1)	11	7
Acceptance/self-consciousness											5	8	1.1 (0.5)	0.9 (1.0)	5	8	4	7	0.9 (0.9)	0.5 (0.6)	4	7	8	12	0.6 (0.2)	7	17
Reading and visual aids																							5	1	0.8 (0.4)	5	1
Household																							7	2	0.3 (0.2)	7	5
Living independent/finances																							8	3	0.2 (0.2)	8	4
Self-care																							8	4	0.3 (0.1)	7	6
Computer skills																							9	6	0.2 (0.2)	8	3
Intimate/romantic relationships																							4	8	0.1 (0.1)	3	10
Peer contact																							6	9	0.4 (0.6)	6	11
Holiday and going out																							11	10	0.4 (0.5)	11	8
Information/regulations																							12	14	0.6 (0.7)	12	13
Applying																							4	16	0.4 (0.3)	4	15
Work																							6	17	0.5 (0.2)	6	16

### Evaluation of the PAI-CY and the PAI-YA

The evaluation forms of parents indicated that over 85% of the parents rated all aspects of the PAI-CY neutral to positive (Table 3). All children and young adults also rated all aspects of the PAI-CY and PAI-YA neutral to positive, except for the duration to fill in the questionnaire. According to participants, the questionnaire was clear, easy to use, and helps to get a first impression before talking to a professional about rehabilitation needs (Table 3). Professionals were more critical regarding the PAI-CY (Table 4). Although over 80% of the professionals were neutral to positive regarding insight into possibilities and limitations of the client using the PAI-CY, about 50% of the professionals were negative about the insight gained of rehabilitation needs and the identification of additional rehabilitation needs using the questionnaires. Furthermore, almost 80% of the professionals rated the questionnaires’ ability to clarify rehabilitation needs negatively (Table 4).

**Table 3:** Evaluation of the PAI questionnaires by parents, children and young adults

Item in evaluation questionnaire	Rating	Number of parents (%)	Number of children/young adults (%)	Statements
Satisfaction with admission procedure	+	16 (67%)	9 (75%)	Questionnaire helps to get a first impression; everything was clear; clear questionnaire; easy to read
	±	8 (33%)	3 (25%) ^a^	
	-	0 (0%)	0 (0%)	
Insight in possibilities for rehabilitation	+	11 (46%)	5 (83%)	No further comments
	±	12 (50%)	1 (17%) ^b^	
	-	1 (4%)	0 (0%)	
Representativeness of problems in daily life	+	7 (29%)	10 (56%)	Nice that there is space for comments; everything was included; some items are very easy/for younger children
	±	14 (58%)	8 (44%)	
	-	3 (13%)	0 (0%)	
Difficulty to choose a response option	+	18 (75%)	13 (72%)	Need a response option between little difficult and very difficult; don’t know some answers
	±	4 (17%	5 (28%)	
	-	2 (8%)	0 (0%)	
Satisfaction with identification of rehabilitation needs	+	14 (58%)	5 (83%)	Makes you think about daily situation
	±	10 (42%)	1 (17%) ^c^	
	-	0 (0%)	0 (0%)	
Satisfaction with duration of completing forms	+	19 (79%)	13 (72%)	No further comments
	±	5 (21%)	2 (11%)	
	-	0 (0%)	3 (17%)	

^a^Only children aged 7–17 years were asked

^b^Only young adults aged 18–25 years were asked

^c^Only children aged 13–17 years were asked

**Table 4:** Evaluation of the PAI questionnaires by professionals

Item in evaluation questionnaire	Rating	Number of professionals (%)	Statements
Insight in rehabilitation needs client	+	2 (12%)	Gives insight in possibilities and limitations, but not in rehabilitation needs
	±	7 (41%)	
	-	8 (47%)	
Insight in possibilities and limitations client	+	8 (47%)	Nice questionnaire to go more into depth; responses to questionnaire give limited information; good to have responses of parent and child
	±	6 (35%)	
	-	3 (18%)	
Estimated perception of client regarding questionnaire	+	12 (71%)	Difficult to fill in for parents because of language use; some items are not applicable because of age; nice to have comment boxes; unclear whether questionnaire should be filled in as using visual aids; response option don’t know is lacking
	±	5 (29%)	
	-	0 (0%)	
Clarification of rehabilitation needs using questionnaire	+	5 (29%)	Unclear what rehabilitation wishes/needs are
	±	0 (0%)	
	-	12 (71%)	
Contribution in development rehabilitation plan	+	0 (0%)	No further comments
	±	17 (100%)	
	-	0 (0%)	
Satisfaction about duration	+	14 (82%)	No further comments
	±	1 (6%)	
	-	2 (12%)	
Correspondence available products to identified needs	+	1 (6%)	No further comments
	±	12 (71%)	
	-	4 (24%)	
Identification of additional rehabilitation needs	+	6 (35%)	Able to identify additional needs that would not have been identified using a semi-structured interview
	±	2 (12%)	
	-	9 (53%)	

Five parents missed some items in the questionnaires, e.g. regarding visual field functioning (0–2 years), energy management (7–12 years), computer/IT and smartphone use (7–12 years), functioning in extracurricular activities (7–12 years) and use of visual aids (13–17 years). Two children indicated they missed items regarding swimming (7–12 and 13–17 years) and one child regarding self-care (13–17 years). Three young adults indicated items that were lacking, e.g. regarding coping with stairs and steps, travelling to unknown locations, and approaching someone you like. Two professionals stated the PAI-CY 3–6 years was more applicable for children aged 3 years than for children aged 6 years, and professionals lacked items regarding reading and writing, behaviour at school, orientation and mobility, and completing tasks in this questionnaire. Two professionals lacked information about the course of pregnancy, childbirth and development in the first years of life.

Parents, young adults and professionals also gave suggestions for the deletion or clarification of some items. Young adults mentioned that some items needed rephrasing to make a distinction between activities performed independently or with others. Furthermore, two items (not standing out as different, and being aware of regulations regarding vehicle driving) were indicated as unclear by young adults. One young adult indicated the domain intimate/romantic relationships was not related to having a visual impairment. Professionals mentioned lack of information on the distance (e.g. looking at something directly) in the PAI-CY 0–2 years and confusion about the interpretation regarding two items (participating in birthday parties, and finding the way in school) in the PAI-CY 3–6 years.

Based on the comments and suggestions of participants and professionals, adaptations were made to the questionnaires with the aim to improve feasibility in practice and acceptability to clients and professionals. In all questionnaires, after each domain an item was added to clarify the rehabilitation needs (Do you have any questions for the rehabilitation centre regarding the topic … or would you like to receive rehabilitation for this?). Furthermore, in all questionnaires items were added, and one and six items were removed in the PAI-CY 3–6 years and the PAI-YA, respectively. Two domains (mobility, and reading and writing) were added in the PAI-CY 3–6 years, and the order of the domains in the PAI-CY 13–17 years and the PAI-YA were slightly adapted based on the comments of participants. For the PAI-CY 13–17 years, adolescents did not like to start with the mobility domain, because items in this domain are generally perceived as most difficult. Therefore, it was chosen to start with leisure time, which was perceived as easier and more fun to start with. For the PAI-YA, the domain acceptance/self-consciousness was perceived as most intrusive, and therefore placed at the end of the questionnaire. In all questionnaires, some items were rephrased, or examples were added or adjusted for clarification purposes. After all additions and deletions, the final PAI-CY 0–2 contains 44 items, the PAI-CY 3–6 years 62 items, the PAI-CY 7–12 years 55 items and the PAI-CY 13–17 tears 58 items, which are respectively 2, 11, 5 and 8 items more than the pilot versions. The final version of the PAI-YA contains 141 items, which is 2 items less than its pilot version. Table 2 provides a summary of the results and Table 5 presents the items that were added to or removed from the questionnaires.

**Table 5:** Items that were added to or removed from the questionnaires (item – domain)

PAI-CY 0–2 years • Looking at something from a distance – Visual attention • Seeing objects in the visual field – Sensorial functioning PAI-CY 3–6 years • Completing tasks – Incentive processing • Being free in movement – Mobility • Cycling – Mobility • Participating in traffic – Mobility • Participating in physical education – Mobility • Playing outdoors – Mobility • Participating in sports – Mobility • ***Talking – Communication*** • Recognizing pictures – Reading and writing • Interest in characters – Reading and writing • Recognizing characters – Reading and writing • Initial reading – Reading and writing • Initial writing – Reading and writing PAI-CY 7–12 years • Gaming – Leisure time • Watching TV – Leisure time • Participating in sports clubs – Leisure time • Making music – Leisure time • Performing hobbies – Leisure time PAI-CY 13–17 years • Gaming – Leisure time • Watching TV – Leisure time • Participating in sports clubs – Leisure time • Making music – Leisure time • Performing hobbies – Leisure time • Brushing teeth – Self-reliance • Going to the bathroom – Self-reliance • Showering/bathing – Self-reliance PAI-YA 18–25 years • Driving a scooter – Mobility • Driving a car – Mobility • Dealing with steps and stairs – Mobility • ***Being aware of regulations regarding driving – Mobility*** • Following a concert or show in a theatre – Leisure time • ***Listening to music – Leisure time*** • ***Brushing teeth – Self-care*** • ***Being informed about new developments – Computer skills*** • ***Knowing what to expect when having a relationship – Intimate/romantic relationships*** • ***Not standing out as different – Acceptance/self-consciousness***	

Items indicated in bold/italic were removed; all other items were added

## Discussion

This pilot study can be considered an initial, critical step in the further development of the PAI-CY and PAI-YA. The questionnaires were modified based on the analysis of responses, the results of the evaluation forms, and the comments and suggestions made by parents, children, young adults and professionals. In general, most parents, children and young adults were satisfied with the content of the PAI and the role the PAI could play in identifying the rehabilitation needs during or prior to an admission at a rehabilitation centre. Professionals were more critical, and stated that the PAI was not able to identify rehabilitation needs, because it provides insight only into the possibilities and limitations. Although this approach is also used in the PAI for adults they are currently working with [14], professionals apparently experienced difficulty in: i) translating the information from the questionnaires to the rehabilitation needs or goals, and ii) selecting a corresponding rehabilitation intervention. Nevertheless, the question that was added to the PAI regarding rehabilitation needs, should help professionals to better identify and translate the possibilities and limitations revealed by the questionnaire into the individual’s rehabilitation needs and goals.

From the evaluation forms and the comments/suggestions made by parents, children, young adults and professionals, the PAI-CY and PAI-YA were generally considered to include the most relevant topics, and most of the items were clear. The instructions for the questionnaires were slightly adapted to clarify how the questionnaire should be administered, and also when the option ‘Not applicable’ should be selected. Furthermore, the language used in the questionnaires was modified to make it easier to understand by parents, children and young adults (e.g. words such as rehabilitation needs/goals were replaced by words such as rehabilitation questions). Based on the suggestions of participants and professionals, minor changes were made to the content of the questionnaires, e.g. the order of the items, the clarity of the items, and the addition/deletion of some items. Although professionals lacked information about the course of pregnancy, childbirth and development in the early years of life, questions about these topics were not added to the PAI-CY/PAI-YA because they do not relate to activities and participation, but rather to health conditions and/or personal factors which are often assessed at an earlier stage.

This study shows that the questionnaires also had important positive qualities, which was confirmed by the evaluation forms of the professionals. The availability of questionnaires for both children and parents allows to compare their responses, a feature highly valued by professionals. However, due to the small sample size, it was not possible to compare agreement/concordance of children and parents; this will be assessed in future studies with a larger sample size. Moreover, the questionnaire prevents important topics from being overlooked by the professional or client, as almost half of the professionals was able to identify additional rehabilitation needs when using the PAI, which would (probably) not have been identified when using an open interview.

The more critical attitude of professionals towards the PAI questionnaires was also reflected in the development of the PAI for adults [14]. For successful implementation of the PAI-CY and PAI-YA in the future, it is important that professionals are satisfied with the questionnaires. Involvement of professionals at an early stage may provide better understanding of the relevance of implementation for themselves and their clients. Furthermore, early involvement of professionals in the implementation process might lead to identification of factors which can influence the future success of implementation [23, 24].

Although pilot studies are generally conducted using qualitative methods such as think-out-loud studies or focus groups [20, 25], this pilot study also aimed to assess the feasibility of the PAI-CY and PAI-YA within the regular admission procedure. Therefore, the PAI-CY was completed prior to (by parents) or during (by children) the admission procedure at a low vision rehabilitation centre. Because most children are referred to a rehabilitation centre and receive an admission at a lower age (generally at 3–6 years), the inclusion of participants in the other age categories took longer than expected. For example, only one PAI-CY 13–17 years was administered during the regular admission, and only one evaluation form from the professional was available for this age category. Due to the lengthy inclusion period, the first author administered the remainder of the questionnaires to children and young adults already receiving care from low vision rehabilitation centres and who were participating in the larger validation study. Consequently, only a limited number of evaluation forms were available from professionals, and only the feasibility of the PAI-CY for the age categories 0–12 years could be confirmed with some conviction. Nevertheless, the procedure for the PAI-CY 13–17 years is comparable to that of the PAI-CY 7–12 years, whereas the procedure of the PAI-YA is comparable to that of the PAI-CY 0–6 years and the PAI for adults [14]. Thus, the results of this study seem to support the feasibility of the PAI-CY and PAI-YA within the regular admission procedure; however, this should be confirmed after the actual implementation, which starts in 2017.

Conducting pilot studies and reporting the results is important, as they are a crucial part of the study design and can provide valuable information for the larger field study and for other researchers [26, 27]. However, due to the small sample size, the results of the present study need to be interpreted with caution. Guidance on sample sizes for pilot studies is rather limited; general guidelines recommend to use about 10 participants [28] or 10% of the final study size [29]. Increasing the sample size of the present pilot study would have extended the study period, which already took longer than expected. Furthermore, it would make it even more difficult to find sufficient participants for the field study; this is already challenging because of the (overall) small size of this population. Moreover, conducting pilot studies with even a few participants can be very informative in terms of assessing feasibility of the procedures, ensuring clarity of the wording, and acceptability of the formats [30].

Clustering of items into domains based on previous studies [4, 5] was supported by the present study, which contributes to the content and face validity of the questionnaires. Although a large number of floor effects was found in all our questionnaires, the deletion of items was done with utmost consideration and reluctance because of the small sample sizes. The identification of floor and ceiling effects requires replication in a larger sample. Our small sample size does not allow to thoroughly evaluate the psychometric properties of the PAI-CY and PAI-YA. Because questionnaire development often employs factor analysis to assess item structure and determine the quality of a questionnaire, we plan to replicate this protocol using factor analysis and item response theory in a larger study population.

In line with the admission procedure for adults, and as a clinical implication for this study, low vision rehabilitation centres in the Netherlands are planning to use the PAI-CY and PAI-YA to make their admission procedures for children/young adults more structured and objective. With additional psychometric testing, the PAI-CY and PAI-YA have great potential for use in identifying rehabilitation needs and contributing to the rehabilitation plan for children and young adults. Professionals can use the questionnaires to get an assessment of a client’s abilities, limitations, and needs and wishes related to participation and activities. Based on this assessment, rehabilitation programmes can be discussed with the client to ameliorate these limitations. The PAI-CY and PAI-YA also have potential to evaluate the effectiveness of rehabilitation when administered pre and post-rehabilitation interventions; improved scores at the end of a rehabilitation programme will be indicative of positive rehabilitation outcomes. Finally, the PAI-CY and PAI-YA could be useful as tools in participation research; e.g. they could be used as an outcome measure in an intervention to increase participation in visually impaired children or young adults.

## Conclusions

This pilot study is an initial, critical step in the validation of the PAI-CY and PAI-YA. Although parents, children and young adults were mostly satisfied with the questionnaires, professionals showed less satisfaction. Nevertheless, the PAI-CY and PAI-YA were considered to include the most relevant topics, and most of the items and response categories were clear to the users. Furthermore, the PAI-CY and PAI-YA seem to be feasible for use in the regular admission procedure of Dutch rehabilitation centres. The adaptations made to the PAI-CY and PAI-YA after the pilot study are likely to improve satisfaction with the content, the clarification of questions, and satisfaction with the questionnaires with regard to compiling a rehabilitation plan. Although face and content validity have been optimized, a larger field study is taking place to further develop the PAI-CY and PAI-YA and assess their properties as a quantitative instrument to reliably assess rehabilitation needs and monitor outcomes of children/young adults with a visual impairment seeking rehabilitation services.

## Abbreviations

ICF: International Classification of Functioning, Disability and Health
PAI: Participation and Activity Inventory
PAI-CY: Participation and Activity Inventory – Children and Youth
PAI-YA: Participation and Activity Inventory – Young Adults
WHO: World Health Organization
